# Body Mass Index and Prognosis of Patients With Stage II/III Gastric Cancer After Curative Gastrectomy: Completion of Perioperative Adjuvant Chemotherapy May Be a Confounding Factor

**DOI:** 10.3389/fonc.2022.899677

**Published:** 2022-06-13

**Authors:** Wei Peng, Jing Dai, Chao-chan Liu, Dian Liu, Hua Xiao

**Affiliations:** ^1^ Gastroenterology and Urology Department II, Hunan Cancer Hospital and the Affiliated Cancer Hospital of Xiangya School of Medicine, Central South University, Changsha, China; ^2^ Department of Lamphoma and Abdominal Radiotherapy, Hunan Cancer Hospital and the Affiliated Cancer Hospital of Xiangya School of Medicine, Central South University, Changsha, China; ^3^ Department of Hepatobiliary and Intestinal Surgery, Hunan Cancer Hospital and the Affiliated Cancer Hospital of Xiangya School of Medicine, Central South University, Changsha, China; ^4^ Department of Gastroduodenal and Pancreatic Surgery, Hunan Cancer Hospital and the Affiliated Cancer Hospital of Xiangya School of Medicine, Central South University, Changsha, China

**Keywords:** body mass index, malnutrition, perioperative adjuvant chemotherapy, gastric cancer, prognosis

## Abstract

**Objective:**

To investigate the association between body mass index (BMI) and overall survival (OS) of patients with stage II/III gastric cancer (GC) after radical gastrectomy, and evaluate the potential influence of perioperative adjuvant chemotherapy (PAC).

**Methods:**

Medical records of 2,510 consecutive stage II/III GC patients who underwent curative resection between November 2010 and December 2020 were retrospectively reviewed. The optimal cutoff value of BMI for OS was determined by X-tile. The independent predictive factors for completeness of PAC were identified using univariate and multivariate logistic regression analyses. Cox regression analyses assessed the association among BMI, completeness of PAC, and OS.

**Results:**

Of the 2,510 patients, 813 cases with BMI < 20.3 kg/m^2^ were classified as belonging in the low BMI group. Further analyses confirmed that low BMI was an independent predictor for incomplete PAC (< 6 cycles, n = 920) and poorer OS (hazard ratio: 1.317, 95% confidence interval: 1.162-1.494, *P* < 0.001), but neo-adjuvant chemotherapy (NAC) was a protective factor. An additive effect was found in those with both low BMI and incomplete PAC, as they had even worse OS. However, in patients with low BMI, completion of PAC (≥ 6 cycles) significantly improved OS, which became comparable to that in the high BMI group (*P* = 0.143).

**Conclusions:**

Low preoperative BMI independently affects completion of PAC and prognosis of patients with stage II/III GC, but completing PAC can compensate for the adverse influence of low BMI on OS. Thus, strategies designed to ensure the completion of PAC, such as NAC and nutritional support, should be further investigated.

## Introduction

Gastric cancer (GC) ranks as the fifth for incidence and fourth for cancer-related deaths globally, with almost 50% occurring in China, and surgery being the only possible curative management so far (1, 2). Unfortunately, only a few patients in China and Western countries are diagnosed at an early stage. Even after undergoing D2 gastrectomy, relapse can be seen within 5 years in about half of those with stage II or III GC (3). To improve overall survival (OS), perioperative adjuvant chemotherapy (PAC), including both pre- and/or postoperatively, has been considered as standard care (4, 5). However, many patients cannot complete all of the allocated courses of chemotherapy for various reasons, such as severe morbidity due to surgery, poor nutritional status or chemotherapy-induced adverse events. In fact, nearly a half of all patients could not complete the planned perioperative management even in recent prospective phase 3 studies (6–8). In one of our previous studies, which included 1,288 stage II/III GC patients (9), only 31.5% completed ≥ 6 cycles of PAC. Further analyses confirmed that completion of at least 6 cycles of PAC was significantly associated with prolonged cancer-specific survival. This was echoed by Noh et al. (3) who reported that those with high relative dose intensity (≥ 6 courses of regimens) had significantly better outcomes in a second *post-hoc* analysis of the well-known CLASSIC study.

There is a growing body of evidence showing that nutritional status not only relates to postoperative morbidity but also oncological outcomes of different types of cancer (9–12). Body mass index (BMI) is a simple and commonly used indicator for assessing nutritional status in the clinic, and several studies have found that low BMI is a significant predictor of poor prognosis in GC patients (13–15). However, some researchers argued that BMI was not related to oncological outcomes (16, 17). While possible explanations for the discordant results include the inconsistency in patient inclusion and BMI classification criteria, the relationship between BMI and prognosis of GC needs further clarification.

Considering that malnutrition also significantly influences chemotherapy-induced adverse events, as well as the completion of PAC (9, 11, 18, 19), we hypothesized that low BMI would be a useful predictor of poor compliance with PAC. Therefore, in this large sample-size study from a high-volume center, we retrospectively assessed the influence of low BMI on the completion of PAC in patients with stage II or III GC. We also explored whether completing PAC could compensate for the adverse impact of low BMI on survival.

## Patients and Methods

### Patients

**Figure 1:**
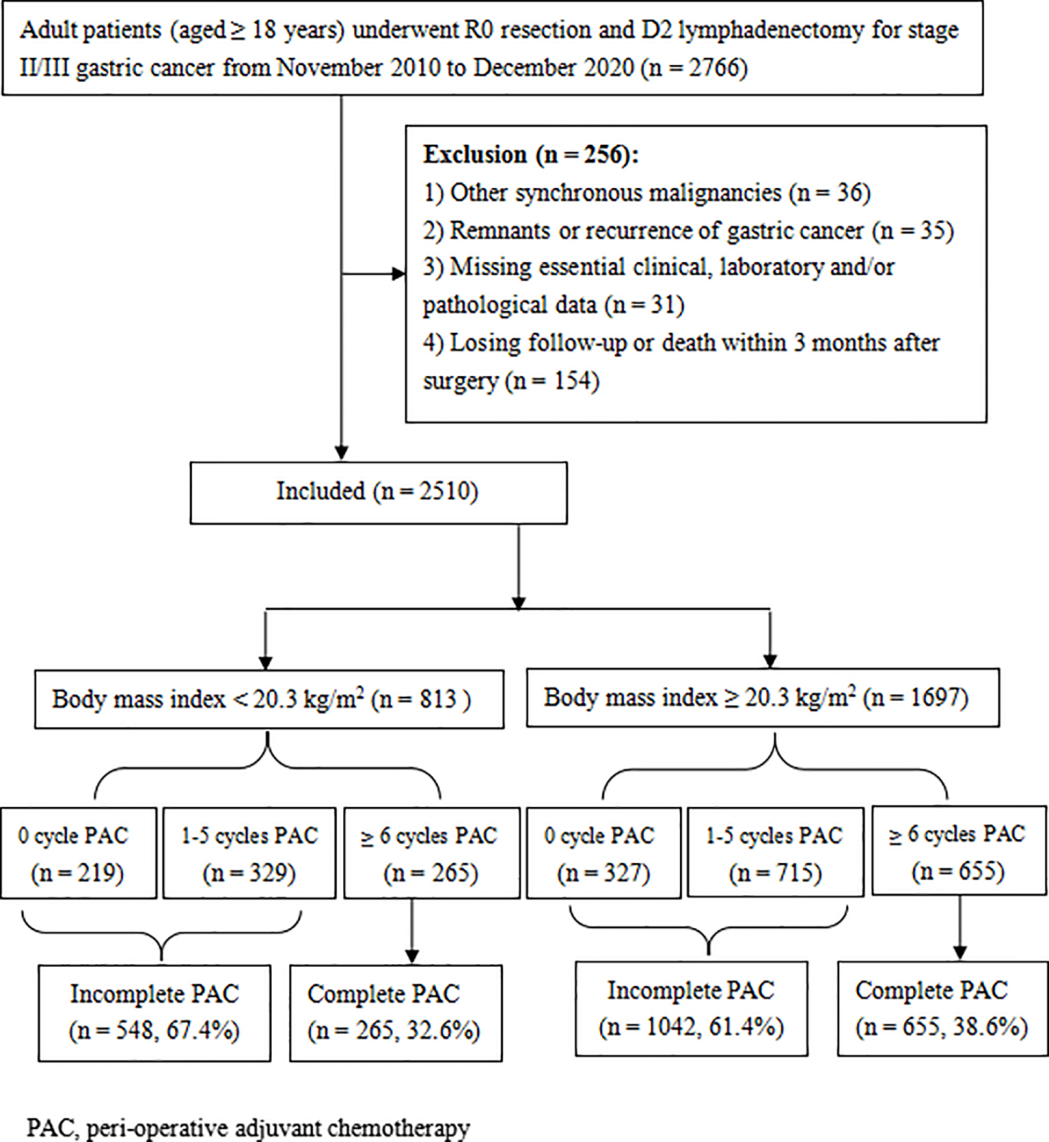
Flow diagram of the present study.

The medical records of all adult patients (≥ 18 years old) who received radical gastrectomy and D2 lymphadenectomy for stage II/III gastric adenocarcinoma in Hunan Cancer Hospital between November 2010 and December 2020 were retrospectively reviewed. The flowchart and exclusion criteria for this study are described in **Figure 1**. This study was conducted in accordance with the guidelines laid down in the Declaration of Helsinki and approved by the ethics committee of the Hunan Cancer Hospital (No. 16 in 2022). Written informed consent for gastrectomy, and the use of their clinical data, has been obtained from all patients prior to surgery.

### Perioperative Management and Follow-Up

Surgeons with sufficient experience performed or supervised all surgeries, according to the Japanese gastric cancer treatment guidelines (5) and staged by the 8th edition of TNM classifications (20). D2 gastrectomy and postoperative adjuvant chemotherapy (AC) was applied to the overwhelming majority of stage II/II GC patients in our institution, as described in our previous studies (9, 21), except for a few patients with cT3-4/N+ diseases, who received 2 to 4 cycles of neo-adjuvant chemotherapy (NAC) before surgery, using platinum and fluorouracil based regimens such as FLOT and XELOX (3, 22). Fluorouracil and platinum-based AC was generally initiated about 3 to 4 weeks following resection and lasted for half a year (3, 22, 23).

Postoperative adverse events were diagnosed within 30 days following gastrectomy and staged by the Clavien-Dindo classification system (21, 24). Patients were followed-up at 1 month after gastrectomy, and once every quarter in the first 2 years, then every half year for the 3rd to 5th year, and once a year thereafter, through December 2021. At each follow-up, patients underwent physical and laboratory measurements, ultrasonography, or a CT scan, and endoscopy was recommended every 2 years.

### Evaluation

Clinicopathological data, including patients’ height and weight, were obtained within 7 days prior to surgery. BMI was calculated as body weight divided by square of height (kg/m^2^), and the cut-off BMI value for OS was selected by X-tile, as described in our previous study (25). For other variables such as age, albumin, and hemoglobin levels, generally accepted or standard clinical thresholds were used. Complete PAC was defined as receiving ≥ 6 cycles of adjuvant chemotherapy perioperatively, because patients receiving less than 6 cycles of PAC had significantly poorer prognosis, according to published literature (3, 9). The evaluated primary outcome was OS, which was defined as the time from gastrectomy until death or the last follow-up, whichever occurred first.

### Statistical Analysis

Continuous variables were compared using Student’s *t*-test or Mann-Whitney U test, and are presented as mean and standard deviation (SD). Categorical data were compared by χ^2^ or Fisher exact test, and are described as numbers (%). Univariate and multivariate regression analyses were utilized to explore factors related to completeness of PAC. The optimal cutoff BMI value for OS was set by X-tile when reaching the maximum χ^2^ log-rank value. The differences of OS in subgroups were compared by Kaplan-Meier curves and log-rank test. Multivariate regression analyses using a forward conditional method were carried out for factors with a *P*-value < 0.05 after univariate analysis. Data was analyzed by SPSS 24.0 software (IBM Corporation, NY, USA) and a two-sided *P* value < 0.05 was considered as statistically significant.

## Results

### Clinicopathological Characteristics

A total of 2,510 consecutive patients were enrolled in this study, and their basic characteristics are presented in **Table 1**. The majority of patients were male (65.6%), with stage III disease (73.0%), who underwent subtotal gastrectomy (71.2%) by open procedure (73.3%). The mean age was 56.1 years (range 19 - 86), with a mean BMI of 21.88 kg/m^2^ (range 13.84 - 37.18), and the mean postoperative duration of hospital stay was 11.7 days (range 3 - 87). Two hundred and sixty-nine patients (10.7%) developed some morbidity within 30 days following resection, defined as Clavien-Dindo grade II or greater.

**Table 1:** Clinicopathological characteristics of the entire cohort, classified by body mass index (BMI) (n =2510).

Variables	BMI < 20.3 kg/m^2^ (n = 813)	BMI ≥ 20.3 kg/m^2^ (n = 1697)`	*P* value
Gender (males)	528 (64.9%)	1119 (65.9%)	0.623
Age (years)	56.23 ± 11.86	56.04 ± 10.40	0.684
Body Mass Index (kg/m2)	18.64 ± 1.23	23.44 ± 2.39	<0.001
Any comorbidities	209 (25.7%)	523 (30.8%)	0.008
Neo-adjuvant chemotherapy	101 (12.4%)	184 (10.8%)	0.243
Pre-operative lymphocyte count (×10^9^/L)	1.66 ± 0.63	1.80 ± 0.67	<0.001
Pre-operative albumin concentration (g/L)	38.12 ± 4.94	39.63 ± 4.58	<0.001
Prognostic nutritional index	46.41 ± 6.43	48.63 ± 6.04	<0.001
Pre-operative hemoglobin (g/L)	112.88 ± 23.88	119.90 ± 25.76	<0.001
Operation method			0.006
Open	625 (76.9%)	1216 (71.7%)	
Laparoscopy	188 (23.1%)	481 (28.3%)	
Type of resection			0.214
Distal subtotal gastrectomy	541 (66.5%)	1187 (69.9%)	
Proximal subtotal gastrectomy	19 (2.3%)	39 (2.3%)	
Total gastrectomy	253 (31.1%)	471 (27.8%)	
Lymph node harvested	20.21 ± 8.17	20.87 ± 8.34	0.063
T stage*			0.071
T1	22 (2.7%)	66 (3.9%)	
T2	83 (10.2%)	169 (10.0%)	
T3	109 (13.4%)	281 (16.6%)	
T4	599 (73.7%)	1181 (69.6%)	
N stage*			0.268
N0	156 (19.2%)	382 (22.5%)	
N1	164 (20.2%)	316 (18.6%)	
N2	211 (26.0%)	418 (24.6%)	
N3	282 (34.7%)	581 (34.2%)	
pTNM stage*			0.025
II	196 (24.1%)	481 (28.3%)	
III	617 (75.9%)	1216 (71.6%)	
Intra-operative blood loss (mL)	203.56 ± 153.78	210.86 ± 146.27	0.250
Operation time (min)	195.74 ± 58.01	204.55 ± 57.80	<0.001
Peri-operative blood transfusion			
Yes	200 (24.6%)	340 (20.0%)	0.009
No	613 (75.4%)	1357 (80.0%)	
Post-operative complications^†^			0.905
Yes	88 (10.8%)	181 (10.7%)	
No	725 (89.2%)	1516 (89.3%)	
Post-operative hospital stays (days)	11.85 ± 5.77	11.64 ± 4.82	0.332
Peri-operative chemotherapy			<0.001
None	219 (26.9%)	327 (19.3%)	
1-5 cycles	329 (40.5%)	715 (42.1%)	
≥6 cycles	265 (32.6%)	655 (38.6%)	

Data are presented as mean ± SD or n (%).

*Tumor stages are based on 8th edition of the Union for International Cancer Control TNM classification.

^†^Defined as Clavien-Dindo grade II or greater.

The cutoff value of BMI for OS was selected as 20.3 kg/m^2^ by X-tile (**Supplementary Figure 1**). A total of 813 patients (32.4%) had a BMI of less than 20.3 kg/m^2^. As shown in **Table 1**, lower BMI was associated with poorer nutritional and immunological status (such as having lower albumin concentration and lymphocyte count), lower hemoglobin level, more advanced tumor stage, and being less likely to receive PAC.

### Predictors for Incomplete PAC

Of a total of 2,510 patients, 1,964 (78.2%) received PAC, but only 920 cases completed at least 6 cycles (36.7%, complete PAC group). In contrast, the remaining 1,590 cases (63.3%) received none or 1 to 5 cycles of PAC, and were considered as incomplete PAC. Not surprisingly, oncological outcomes were significantly better in patients receiving complete PAC compared with those patients who received none or 1 to 5 cycles of PAC (the mOS were 108, 54, and 36 months, respectively, *P* < 0.001), regardless of having stage II or III disease (**Supplementary Figure 2**).

The clinicopathological variables were retrospectively analyzed to evaluate their potential influence on compliance with PAC. Univariate analyses revealed that age, BMI, albumin levels, NAC, operation procedure, operative time, perioperative blood transfusion, and tumor stage potentially affect the completeness of PAC (all *P* < 0.05). Further multivariate analyses confirmed that PAC completeness was negatively impacted only by older age (≥ 65 years), lower albumin level (< 35 g/L), and lower BMI (< 20.3 kg/m^2^), while NAC was identified as a protective factor (**Table 2**). In fact, 38.6% (655/1697) of patients with BMI ≥ 20.3 kg/m^2^ received at least 6 cycles of PAC, which was significantly better than that in the low BMI group (32.6%, 265/813, *P* = 0.003) (**Figure 1**).

Table 2Association between clinicopathological characteristics and completeness of peri-operative adjuvant chemotherapy (PAC, ≥ 6 cycles) after gastrectomy for stage II/III gastric cancer (n = 2510).

**Table:** 

Variables	Complete PAC (n = 920)	Incomplete PAC (n = 1590)	*P* value
Sex (Male/Female)	592/328	1055/535	0.308
Age (years) ≥ 65/<65	119/801	469/1121	<0.001
Body mass index (kg/m2) ≥ 20.3/<20.3	655/265	1042/548	0.003
Hemoglobin (g/L) ≥ 100/<100	205/715	397/1193	0.129
Comorbidity; yes/no	247/673	485/1105	0.052
Albumin level (g/L) ≥35/<35	802/117	1247/340	<0.001
Neo-adjuvant chemotherapy; yes/no	183/737	102/1488	<0.001
Extent of gastric resection; subtotal/total	654/266	1132/458	0.954
Operation time (min) ≥ 240/<240	251/668	351/1237	0.003
pTNM stage^†^; III/II	707/213	1126/464	0.001
Peri-operative blood transfusion; yes/no	176/744	364/1226	0.027
Post-operative complications^‡^; yes/no	86/834	183/1407	0.092

**Table:** Multivariate analysis of possible predictors for completeness of peri-operative adjuvant chemotherapy (PAC, ≥ 6 cycles) after gastrectomy for stage II/III gastric cancer (n = 2510).

Variables	Odds Ratio [OR]	95% Confidence Interval [CI]	*P* value
Age ≥ 65 years	2.618	2.090-3.279	<0.001
Albumin level < 35 g/L	1.658	1.307-2.103	<0.001
Neo-adjuvant chemotherapy, yes	0.286	0.219-0.372	<0.001
Body mass index < 20.3 kg/m^2^	1.270	1.055-1.529	0.011

†Tumor stages are based on 8th edition of the AJCC TNM classification.

‡Defined as Clavien-Dindo grade II or greater.

### Predictors for OS

During a median follow-up of 28 months (range 4 - 132), 1,050 of 2,510 patients (41.8%) died, with a mOS of 63 months. Death was more common in patients with BMI < 20.3 kg/m^2^ (48.3%, 393/813) compared with that in patients with higher BMI (38.7%, 657/1697, *P* < 0.001).

Univariate analyses revealed that age, BMI, American Society of Anesthesiologist (ASA) score, albumin level, operation procedure, length of operation, intra-operative blood loss, type of resection, TNM stage, perioperative blood transfusion, postoperative complications, and PAC were potentially related to OS (all *P* < 0.05). After multivariate Cox regression analyses, BMI < 20.3 kg/m^2^ was confirmed to adversely affect OS (hazard ratio (HR): 1.317, 95% confidence interval (CI): 1.162 - 1.494, *P* < 0.001). In contrast, complete PAC (≥ 6 cycles) was confirmed to be a protective variable (HR: 0.527, 95% CI: 0.458 - 0.607, *P* < 0.001) (**Table 3**).

**Table 3:** Univariate and multivariate analyses of prognostic factors for overall survival following radical gastrectomy of stage II/III gastric cancer (n = 2510).

Variables	N	Median OS time (months)	UV*P* value	MVHR (95% CI)	MV*P* value
Gender			0.907		
Male	1647	63.0			
Female	863	65.0			
Age (years)			<0.001		0.012
≥65	588	44.0		1.198 (1.041-1.378)	
<65	1922	69.0		Reference	
Body mass index (kg/m^2^)			<0.001		<0.001
<20.3	813	44.0		1.317 (1.162-1.494)	
≥20.3	1697	77.0		Reference	
ASA score			<0.001		0.051
≥3	209	40.0			
<3	2301	70.0			
Comorbidities			0.226		
Yes	732	79.0			
No	1778	61.0			
Hemoglobin (g/L)			0.311		
≥100	1908	65.0			
<100	602	61.0			
Albumin level (g/L)			0.019		0.862
≥35	2049	67.0			
<35	457	54.0			
Operation procedure			0.008		0.433
Open	1841	60.0			
Laparoscopy	699	68.0			
Operation time (min)			<0.001		0.004
≥240	602	38.0		1.233 (1.068-1.424)	
<240	1905	73.0		Reference	
Intra-operative blood loss (mL)			<0.001		0.037
≥300	522	41.0		1.168 (1.009-1.351)	
<300	1987	70.0		Reference	
Type of resection			<0.001		<0.001
Total gastrectomy	724	28.0		1.739 (1.579-2.047)	
Sub-total gastrectomy	1786	91.0		Reference	
pTNM stage^†^			<0.001		<0.001
III	1833	41.0		2.983 (2.498-3.562)	
II	677	NA*		Reference	
Peri-operative blood transfusion			<0.001		0.302
Yes	540	42.0			
No	1970	70.0			
Post-operative complication^‡^			<0.001		0.016
Yes	269	34.0		1.260 (1.044-1.520)	
No	2241	68.0		Reference	
Peri-operative adjuvant chemotherapy (cycles)			<0.001		<0.001
≥6	920	NA*		0.527 (0.458-0.607)	
<6	1590	45.0		Reference	

ASA, American Society of Anesthesiologist; OS, overall survival; CI, confidence interval; HR, hazard ratio; UV, univariate analysis; MV, multivariate analysis; NA, not available.

*The median overall survival time has not reached during the follow-up.

^†^Tumor stages are based on 8th edition of AJCC TNM classification.

^‡^Defined as Clavien-Dindo grade II or greater.

### Relationship Among BMI, PAC, and OS

**Figure 2:**
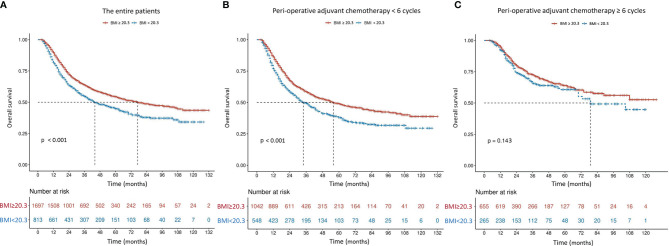
Overall survival curves in 2,510 patients who underwent curative resection for stage II/III gastric cancer classified by body mass index [BMI, < 20.3 or ≥ 20.3 kg/m^2^ **(A)**] and further stratified by perioperative adjuvant chemotherapy [< 6 or ≥ 6 cycles **(B, C)**]. The differences of overall survival in subgroups were compared by log-rank test.

**Figure 3:**
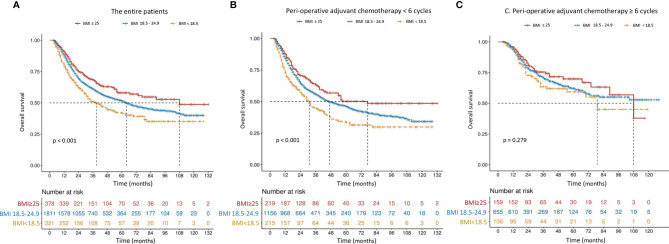
Overall survival curves in 2,510 patients who underwent curative resection for stage II/III gastric cancer classified by body mass index [BMI, < 18.5, 18.5-24.9 or ≥ 25.0 kg/m^2^ **(A)**] and further stratified by perioperative adjuvant chemotherapy [< 6 or ≥ 6 cycles **(B, C)**]. The differences of overall survival in subgroups were compared by log-rank test.

The mOS in patients with higher BMI (≥ 20.3 kg/m^2^) was 77 months, which was significantly better than 44 months in those with lower BMI (*P* < 0.001) (**Figure 2A**). Although the difference was still significant among patients receiving incomplete PAC (56 months vs. 34 months, *P* < 0.001, **Figure 2B**), mOS became comparable in patients who received at least 6 cycles of PAC, regardless of BMI (not available vs. 79 months, *P* = 0.143, **Figure 2C**). Results were similar when patients were classified to 3 subgroups according to the World Health Organization (WHO) classification of BMI (< 18.5, 18.5-24.9 and ≥ 25.0 kg/m^2^) (**Figure 3**).

**Figure 4:**
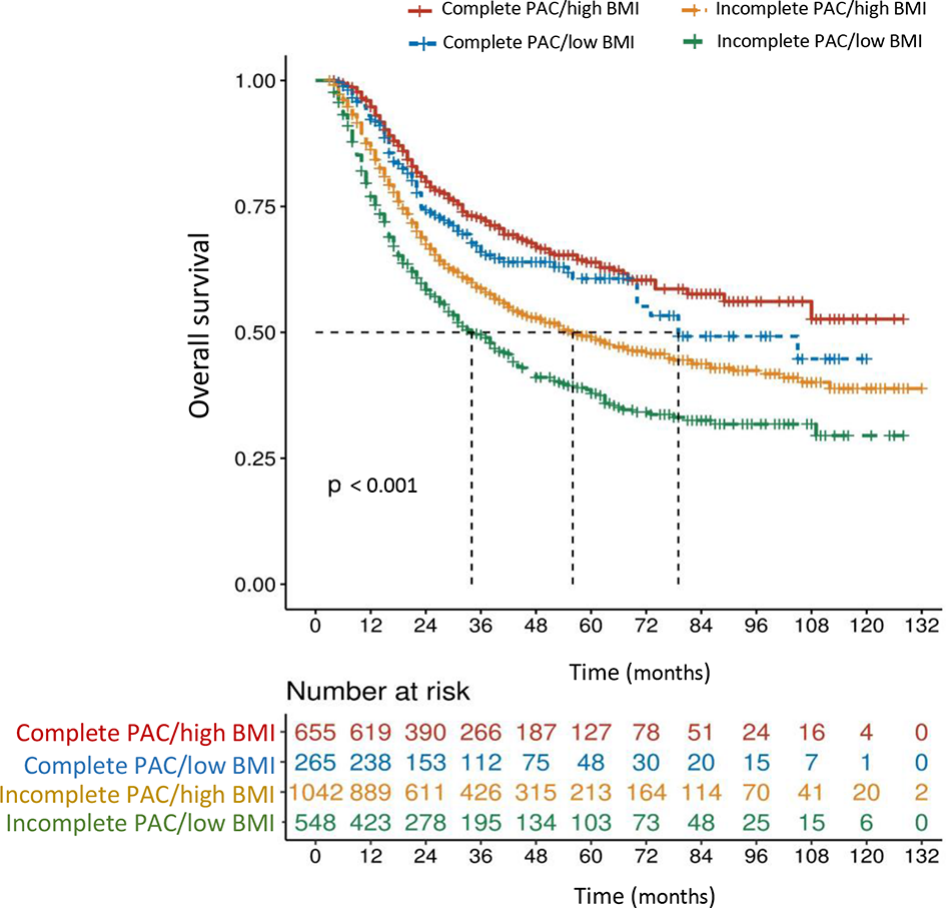
Overall survival curves in 2,510 patients who underwent curative resection for stage II/III gastric cancer classified by the body mass index (BMI < 20.3 or ≥ 20.3 kg/m^2^) and perioperative adjuvant chemotherapy (PAC) (low BMI defined as < 20.3 kg/m^2^, high BMI defined as ≥ 20.3 kg/m^2^, incomplete PAC defined as < 6 cycles, complete PAC defined as ≥ 6 cycles). The differences of overall survival in subgroups were compared by log-rank test.

The mOS in patients with high BMI (≥ 20.3 kg/m^2^) who received complete PAC (≥ 6 cycles, n = 655) did not reach statistical significance when compared with that in patients with low BMI and complete PAC (79 months, n = 265, *P* = 0.145), but was significantly better than that in patients with high BMI and incomplete PAC (56 months, n = 1,042, *P* < 0.001), and in patients with low BMI and incomplete PAC (34 months, n = 548, *P* < 0.001) (**Figure 4**). In addition, a synergistic effect was identified in the incomplete PAC/low BMI group, when using the incomplete PAC/high BMI group as a reference (HR: 1.384, 95% CI: 1.199-1.599, *P* < 0.001).

## Discussion

In the present large cohort study including 2,510 consecutive patients from a high-volume center, we revealed that approximately one-third of the patients (32.4%) with stage II/III GC had poor nutritional status (defined as BMI < 20.3 kg/m^2^ in this study), which was consistent with previous studies (13, 14). For the first time, low pre-operative BMI was confirmed as a simple but independent risk factor for poor compliance with PAC. Additionally, low BMI was identified to be associated with poor oncological outcomes, confirming the results of previous studies (13–15). However, further analyses found that if patients completed at least 6 cycles of PAC, then survival time became independent of BMI, regardless of whether they were classified by 20.3 kg/m^2^ or the WHO classification. Our findings suggest that completion of PAC might be a confounding factor between the relationship of low BMI and poor oncological outcomes of stage II/III GC. To compensate for the negative influence of low BMI on prognosis, strategies aimed to ensure the completion of PAC should be further investigated.

Malnutrition is commonly seen in patients with locally advanced GC because of decreased appetite and occasional pyloric obstruction. There is a growing body of evidence showing that nutritional status is significantly associated with prognosis in various malignancies, including GC (9–15). Potential mechanisms include suppression of the immune system, which plays an inevitable role in eliminating cancer cells and preventing metastasis (9, 12, 24). BMI can be easily calculated and is a widely used indicator to assess patients’ nutritional status in the clinic. Although some studies have explored the influence of BMI on the prognosis of GC, their conclusions are still controversial (13–17, 26–28). In a retrospective study containing 1,210 stage I to III GC patients treated with D2 gastrectomy, prognosis was significantly worse in patients with low BMI (< 18.5 kg/m^2^) than in patients with normal (18.5 - 24.9 kg/m^2^) or high (≥ 25.0 kg/m^2^) BMI, after propensity score matching for tumor depth, lymph node metastasis, and tumor stage (26). In contrast, another study analyzing 947 stage I to III GC patients, concluded that both OS and cancer-specific survival were similar among the 3 groups (BMI < 25, 25-30, and > 30 kg/m^2^) (27). Meanwhile, Kim and colleagues (28) reported that preoperative low BMI (< 18.5 kg/m^2^) significantly impacted recurrence and survival in those with stage I/II GC but lost its significance in those with stage III/IV disease. Possible explanations for these discordant findings were inconsistent patient inclusion and BMI classification criteria and relatively small sample sizes. Moreover, stage I GC patients rarely develop severe malnutrition, usually require no chemotherapy, and have significantly better outcomes, thus it seems difficult to define the influence of nutritional status on prognosis for these patients. Additionally, stage IV GC patients with metastatic disease, who usually cannot undergo curative gastrectomy and experience extremely dismal prognoses, were included in some previous studies (28).

Park et al. (14) retrospectively analyzed 1,868 stage II/III GC patients receiving gastrectomy, and concluded that pre-operative underweight (BMI < 18.5 kg/m^2)^ was a significant predictor of recurrence, along with age and TNM stage (*P* < 0.001). In contrast to our findings, underweight patients seemed more likely to receive adjuvant chemotherapy, and the prognosis for these patients was still significantly poorer, regardless of chemotherapy. However, the exact number of cycles of chemotherapy has not been described and thus, the potential impact of BMI on completeness of PAC and the influence of dose intensity on prognosis could not be evaluated. To the best of our knowledge, the potential influence of completeness of PAC on the relationship between BMI and prognosis of GC has never been evaluated to date. Therefore, we conducted this study to investigate the relationship among BMI, PAC, and prognosis of patients with stage II or III GC, by utilizing the data of 2,510 patients.

Although PAC has been considered as the most effective strategy to improve oncological outcomes of those with stage II or III GC, in addition to radical gastrectomy, only 36.6% of patients completed at least 6 cycles of PAC. Further analyses found that older age and poorer nutritional status (low BMI and albumin concentration) adversely affected the compliance with PAC, which was consistent with previous studies (9, 18, 29). Seo et al. (19) reported that older age significantly increased chemotherapy-induced grade 3/4 non-hematological toxicity, but low BMI and hypoalbuminemia were independently associated with grade 3/4 hematological adverse events, which may explain our findings, at least in part. A randomized clinical trial evaluated the effects of post-discharge oral nutritional supplements (ONS) on nutritional outcomes and chemotherapy tolerance in patients at nutritional risk following resection for GC. The 171 patients receiving ONS had significantly higher BMI and less chemotherapy modifications following 3 months of intervention, compared with those who received dietary advice alone (n = 166) (30). These findings suggest that postoperative nutritional supplementation is not optional but a prerequisite, especially in those with malnutrition.

The mOS of patients with low BMI increased from 34 to 79 months when they received ≥ 6 cycles of PAC, which was comparable to those with high BMI (*P* = 0.143, **Figure 3C**). It seems that complete PAC can compensate for the adverse impact of low BMI on survival. Our findings strongly support the importance of complete and adequate PAC, especially in those with low BMI. Our analyses confirmed that NAC was a protective strategy for complete PAC. This was echoed by Li et al. (31), who conducted a retrospective study of 206 patients and concluded that NAC was a significant protective factor to ensure that patients complete all intended multimodal therapy, in order to negate the adverse influence of postoperative complications on oncological outcomes. Thus, in order to complete PAC and thereby mitigate the negative influence of low BMI on oncological outcomes, it may be preferable to perform chemotherapy preoperatively, instead of postoperatively. However, further prospective studies should be conducted to clarify this hypothesis.

Although the present study has some interesting findings, it also has several limitations. First, it was a retrospective study, thus the exact reasons for termination of chemotherapy could not be determined. For example, the type of medical insurance and potential economic burden may act as confounders. In addition, some patients might experience early recurrence (adjuvant chemotherapy was usually performed within 6 months following surgery) and thus terminated PAC or received palliative chemotherapy, which may impact the reliability of our conclusions. Second, it seemed that the median follow-up of 28 months was relatively short, and as a result, later recurrence and death of patients could not be analyzed. Third, patients generally received fluorouracil and platinum based regimens for PAC in our institution. Whereas different combinations have been used, such as S-1 alone, XELOX, SOX, ECF, FLOT, and oxaliplatin plus fluorouracil/leucovorin, given the long duration over 10 years of our study (21). The convenience, incidence, and grade of chemotherapy-caused adverse events induced by different chemotherapy regimens might also affect the compliance with PAC. Last, only 11.4% of all patients received NAC, which was significantly less than in Western countries (32), given that AC following D2 gastrectomy was recommended in Asia (5, 21). As a result, this may influence the generalizability of our findings. Notwithstanding the limitations, this is the first study to evaluate the association among BMI, PAC, and oncological outcomes of patients with stage II or III GC following curative resection, based on a large number of patients.

In conclusion, the present study confirmed that preoperative low BMI is an independent predictor for incompleteness of PAC and poorer oncological outcomes in those with stage II or III GC, but complete PAC could compensate for the negative influence of low BMI on oncological outcomes. Thus, strategies to improve compliance with PAC, such as NAC and nutritional supplements, should be further investigated.

## Data Availability Statement

The original contributions presented in the study are included in the article/**Supplementary Material**. Further inquiries can be directed to the corresponding author.

## Ethics Statement

The studies involving human participants were reviewed and approved by the ethics committee of Hunan Cancer Hospital. The patients/participants provided their written informed consent to participate in this study.

## Author Contributions

HX designed the research, analyzed the data, and critically revised the manuscript. WP collected part of the data, and grafted the manuscript. JD, C-CL and DL collected part of the data. All authors contributed to the article and approved the submitted version.

## Funding

This study was supported by Hunan Cancer Hospital Climb Plan (No. 2020NSFC-A004), Hunan Provincial Natural Science Foundation of China (No. 2020JJ5339), the Science and Technology Foundation of Changsha (No. kq1907125).

## Conflict of Interest

The authors declare that the research was conducted in the absence of any commercial or financial relationships that could be construed as a potential conflict of interest.

## Publisher’s Note

All claims expressed in this article are solely those of the authors and do not necessarily represent those of their affiliated organizations, or those of the publisher, the editors and the reviewers. Any product that may be evaluated in this article, or claim that may be made by its manufacturer, is not guaranteed or endorsed by the publisher.

## Supplementary Material

Supplementary Figure 1 X-tile analysis of overall survival performed using patients’ data to determine the optimal cut-off value for body mass index (BMI). In the left panels, the X-axis represents all potential cut-off values from low to high (left to right) that define a low subset, whereas the Y-axis represents the cut-off values from high to low (top to bottom) that define a high subset. Red coloration of a cut-off value indicates an inverse correlation with time to death, and the green coloration represents direct associations. The optimal cut-off value highlighted by the black circles in the left panels is shown in the histogram of the entire cohort (middle panels). Kaplan-Meier plots are displayed in the right panels, where blue represents the low subgroup and gray represents the high subgroup. The optimal cut-off values for BMI was 20.3 kg/m2, with maximum χ2 long-rank value of 26.5864 and minimum *P* value < 0.0001.

Supplementary Figure 2 Overall survival curves in 2,510 patients who underwent curative resection for stage II/III gastric cancer classified by completeness of perioperative adjuvant chemotherapy (PAC, 0, 1-5 or ≥ 6 cycles), and further stratified by tumor stage (stage II or III). The differences of overall survival in subgroups were compared by log-rank test.
