# Supplementary figures and images for: Body Mass Index and Prognosis of Patients With Stage II/III Gastric Cancer After Curative Gastrectomy: Completion of Perioperative Adjuvant Chemotherapy May Be a Confounding Factor
Source: Front Oncol. 2022 Jun 13;12:899677. doi: 10.3389/fonc.2022.899677 (PMC9234174; doi:10.3389/fonc.2022.899677)

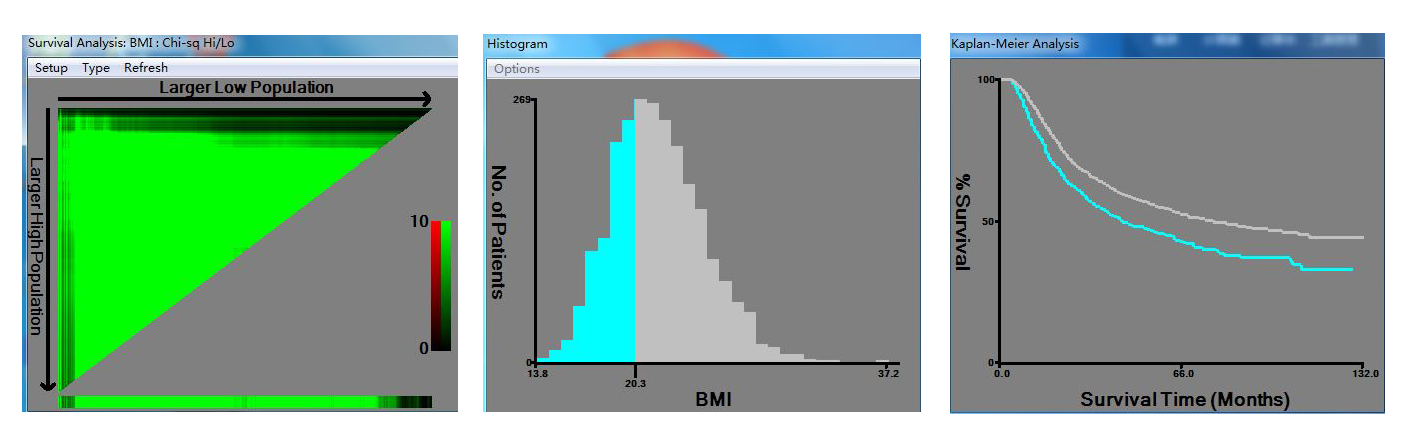

Supplement: Supplementary Figure 1 — X-tile analysis of overall survival performed using patients’ data to determine the optimal cut-off value for body mass index (BMI). In the left panels, the X-axis represents all potential cut-off values from low to high (left to right) that define a low subset, whereas the Y-axis represents the cut-off values from high to low (top to bottom) that define a high subset. Red coloration of a cut-off value indicates an inverse correlation with time to death, and the green coloration represents direct associations. The optimal cut-off value highlighted by the black circles in the left panels is shown in the histogram of the entire cohort (middle panels). Kaplan-Meier plots are displayed in the right panels, where blue represents the low subgroup and gray represents the high subgroup. The optimal cut-off values for BMI was 20.3 kg/m2, with maximum χ2 long-rank value of 26.5864 and minimum P value < 0.0001. [file Image_1.tif]

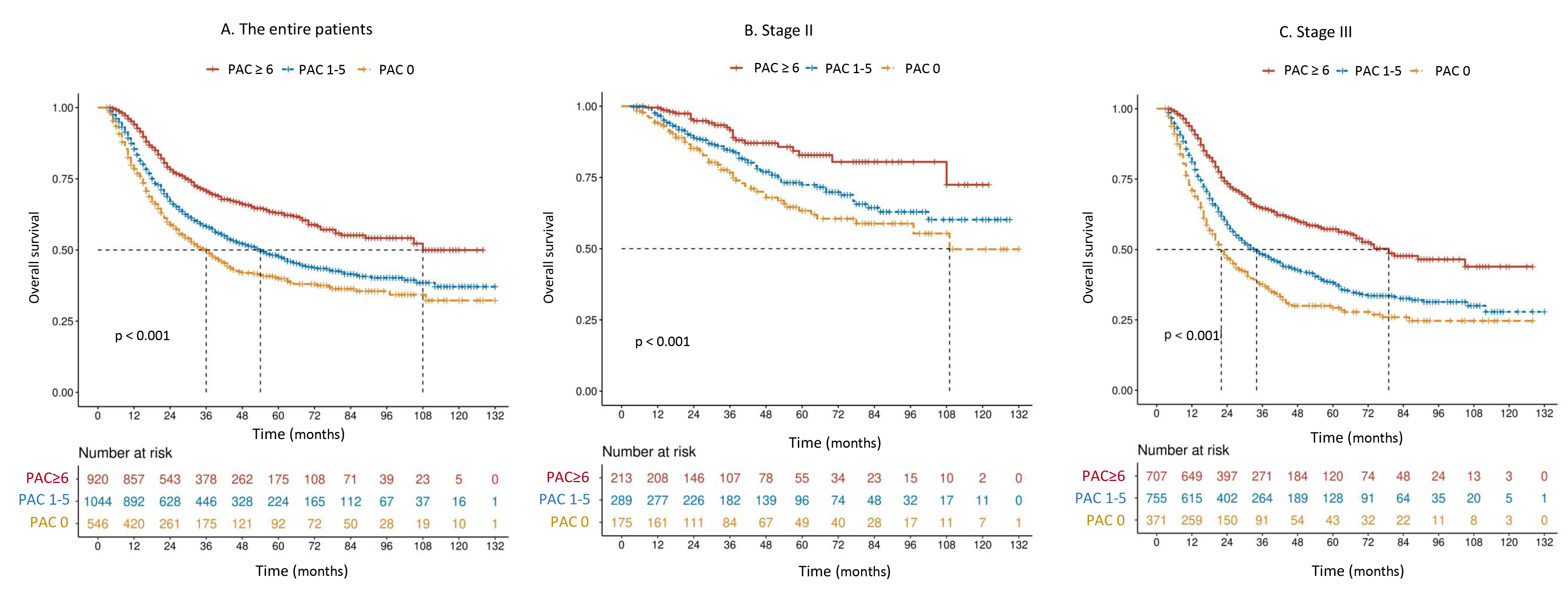

Supplement: Supplementary Figure 2 — Overall survival curves in 2,510 patients who underwent curative resection for stage II/III gastric cancer classified by completeness of perioperative adjuvant chemotherapy (PAC, 0, 1-5 or ≥ 6 cycles), and further stratified by tumor stage (stage II or III). The differences of overall survival in subgroups were compared by log-rank test. [file Image_2.tif]
